# I. Tendo-Vaginitis of the Metacarpo-Phalangeal Sheath. II. Paint (Lead) Poisoning in Cows

**Published:** 1900-06

**Authors:** L. Van Es

**Affiliations:** Mobile, Ala.


### I. TENDO-VAGINITIS OF THE METACARPO-PHALANGEAL SHEATH. II. PAINT (LEAD) POISONING IN COWS.

By L. Van Es, M.D., V.S.,

MOBILE, ALA.

Case I.—This case occurred in a young mare of moderate size and value, and presented itself for treatment about one year ago.

Examination revealed a bilateral fluctuating swelling just above the fetlock. Considerable tenderness on pressure and a pronounced supporting leg lameness.

My diagnosis was tendo-vaginitis of the metacarpo-phalangeal sheath.

At first my treatment consisted of cold applications, alternated by the use of a rubber bandage. The slight improvement brought about by this treatment was not permanent, and the animal was soon back for treatment.

The enlargement having increased to a considerable extent, I now tried to get relief from aspirating, again followed by the applications of the rubber bandage, all to no avail.

As the animal returned lamer than ever, I used a smart vesicant, but with no material result. After the skin had recovered from the effect of the blistering I applied the thermocautery, drawing several oblique lines over the affected part.

No improvement, however, followed this treatment. I now lost sight of the mare for some time, but finally she was brought to me again by a different owner.

The tendon sheath was now enormously enlarged, presenting a "doughy" sensation on pressure. The enlargement, which at first was bilateral, now extended around posteriorly. The animal was extremely lame, and did not attempt to bear weight on the leg.

I now began to consider the advisability of a median neurectomy, but as the enlargement was of such proportions as to constitute quite an impediment to locomotion, I concluded to open the sheath, to remove its contents, and, if practicable, to remove also the diseased part of the sheath. The owner consented readily to any operative procedure I might choose.

The leg was prepared for operation as follows: The hair was closely clipped and scrubbed with brush and soap, washed with hot water, and rinsed with methyl alcohol. The leg was now wrapped in absorbent cotton, bandaged, and this whole dressing kept saturated with a 1 per cent. solution of bichloride of mercury until the following day, when the operation took place. The patient was secured on the operating-table, the dressings removed, and the parts again subjected to an irrigation with a sublimate solution.

I now made an incision, about five inches in length, in the median line on the posterior surface of the limb.

This incision opened the enlarged sheath, which was found to be tightly filled with a large fibrinous clot and a serohemorrhagic exudate, which I removed by scooping out the cavity with my index finger. The clot and liquid exudate about filled a one-quart jar.

The sheath itself was quite thickened and infiltrated with serum.

The removal of the sheath was done by means of a sharp curette, and included everything between its upper limit down as far as the sesamoid pulley or gliding surface, where no evidence of disease was noticeable.

The bleeding was controlled by pressure, the cavity irrigated with a bichloride of mercury solution and packed with sterile iodoform gauze. The edges of the wound were approximated with heavy silk stitches, the parts covered with a layer of gauze, wrapped in absorbent cotton, and the whole enclosed in a neat spica bandage. This first dressing remained until the bandage became stained with wound secretion (three days), when it was removed.

The wound was again irrigated with a sublimate solution and dressed as before.

This dressing was repeated every four or five days, except the packing of the cavity, which was discontinued after the third dressing. The wound healed rapidly, and after two weeks the lameness commenced to improve. Five weeks after the operation the patient was discharged, and as no lameness remained the median neurectomy had not to be resorted to.

There remained somewhat of an enlargement, but it did not cause any trouble to the animal and only slightly disfigured the limb.

CASE II.—On a Friday morning the owner of two Jersey cows and one yearling requested me to attend one of his cows, which had on that morning refused her food and showed other signs of sickness.

On examination the cow presented some nervousness, slight abdominal pain (shown by periodical uneasiness and kicking against her abdomen), muscular twitching at various parts, a constant grinding with her teeth, and frothing from the mouth. Her pulse was small and rapid, temperature and respirations normal, evacuation from the bowels of normal quantity and consistency, but of an extremely dark color.

As far as I could learn from the attendant, the animal had passed urine as usual. The reaction of the pupil to light was normal. As for some time I had seen painters working about the house, I asked if it could have been possible for this cow to have licked paint or have eaten something with paint on it. Even on close questioning this was answered in the negative.

Being thus kept in the dark about my case, I put her on symptomatic treatment, consisting of a mixture of chloral hydrate and bromide of potassium, dissolved in water. I gave this in order to relieve the nervous symptoms and the muscular twitchings.

On my second visit (in the afternoon of the same day), I found my patient in the same condition, but evacuations from the bowels had ceased since morning.

I prescribed one pound of Glauber salts and continued the other treatment. I again questioned the attendant about the paint, but with the same result.

On the following morning (Saturday) the cow did not show any marked change, although the nervousness had largely subsided and the muscular twitchings were now confined to a jerky movement of the abdominal muscles. Constipation persisted, with the exception of two or three scant and solid evacuations.

After examining my patient my attention was called to the other cow and the yearling. Much to my surprise those animals presented about the same symptoms as cow No. 1. The yearling, in addition to these, proved to be blind, with a dilated pupil, totally indifferent to light.

I now became more than ever suspicious of lead-poison, and informed the owner that in spite of the apparent impossibility of the cows being able to get paint, I was reasonably certain that they had got some, nevertheless. I gave each cow and the yearling a good dose of sodium sulphate, and put them on the chloral and bromide mixture.

My afternoon visit disclosed no change in the patients.

On Sunday morning, while being given some oil to relieve the constipation, cow No. 1 suddenly collapsed and died.

Through an unavoidable circumstance I was deprived of the opportunity of an autopsy in this case.

The remaining animals were also given some oil, and by evening they had copious, soft discharges from the bowels.

Their nervous symptoms having become milder, I ordered the sedative to be given at greater intervals.

During the following days most symptoms disappeared, except a total loss of appetite in both animals and the amblyopia and chorea-like movements of the anterior limbs of the yearling.

Those movements, especially noticeable immediately after rising, consisted of a jerky, swinging movement of the entire front limbs alternately, the movements soon ceasing and some stiffness remaining.

The cow gradually began to show signs of improvement, the appetite returned; after administration of a bitter stomachic the nervous symptoms disappeared, and within a short time she was discharged from treatment.

The yearling lost her involuntary muscular movements by degrees, and in the course of several days regained her appetite. As the amblyopia, however, persisted I put her on daily doses of iodide of potassium and strychnine, which had little or no influence on this condition.

During one of my visits I took occasion to walk into the cow lot and to examine a pile of rubbish which I found there. In this pile I found several rags which had been used by the painters to wipe some of their utensils or work, and were saturated with red paint. This discovery satisfied me as to the true nature of my cases.

The condition of the calf remained the same until about three weeks after the first signs of sickness presented themselves, when she was suddenly taken by a hemiplegia and died the following day.

On the post-mortem examination the following conditions presented themselves : A marked pallor of the skeletal muscles, small hemorrhages in the subcutaneous connective tissue and in the abdominal viscera. Digestive organs without any change ; their contents normal in quantity, consistency, and color.

Liver of a grayish-white color, normal in size, very soft and doughy, leaving, on incision, fat globules on the knife.

Spleen normal in appearance.

Kidneys somewhat smaller than I would consider normal, white in color, very soft and friable, giving the impression of having undergone a mucoid degeneration. On section small hemorrhages are to be noticed in the cortical portion.

Bladder about half-full of highly colored limpid urine, which, much to my regret, I neglected to analyze.

Lungs normal ; heart very pale (fatty degeneration), but otherwise normal. The larger bloodvessels showed no change. On opening the cranial cavity I found numerous small hemorrhagic spots in the meninges, while in the brain substance there were noticeable only a small number of exceedingly small hemorrhages. Owing to lack of time I failed to take out the spinal cord.

Specimens taken from the cerebrum, kidneys, and liver showed on microscopical examination the following lesions : Sections from the cerebral cortex and stained according to Nissl's method showed the cells to have taken the stain very diffusely ; no differentiation of nucleus, nucleolus, or pigment body had taken place. I am, however, not prepared to say whether this condition was due to a pathological change in the neurons or whether this was due to a slight deviation from the method as laid down by Nissl. This deviation consisted in placing the fresh specimen in a formalin solution instead of strong alcohol, the formalin being the only fixing agent I happened to have along.

Liver and kidney specimens were treated as follows : Fixed in formalin dehydrated in 60, 80, and 96 per cent. alcohol, embedded in collodion, sections cut with microtome, stained in alum, carmine, cleared with carbol xylol, and mounted in Canada balsam.

Sections of liver, when examined with the naked eye or a low power objective (2/III), present a moderate number of highly stained specks or patches, very similar but smaller than those seen in miliary tubercle. When studied under a higher power (1/IV) these patches prove to be masses of leucocytes in the interlobular tissue, indicating an interstitial hepatitis.

At various points I also noticed a beginning of an interlobular connective tissue proliferation. The walls of the interlobular vessels are more or less sclerotic. The hepatic cells show different stages of a granular and fatty degeneration, but not to such an extent as the gross appearance of the organ would warrant one to anticipate.

The nuclei, although granular in appearance, still have retained their affinity for the stains to a greater or less degree At some points, however, I notice small patches where all cellular structure has disappeared, nothing remaining but a granular or fatty débris.

Sections of kidney, stained and mounted as indicated above, give a picture of a pronounced parenchymaotus degeneration. Especially in the cortical portion of the organ one can only with the greatest difficulty find sections of tubules in which the epithelium has remained intact so as to show cellular structure. Such cells, however, also show the beginning of degenerative changes.

In almost all the convoluted tubules the epithelium has completely broken down, the débris filling the lumen, and nothing remaining but the thin casement membrane.

In a great number of places an interstitial inflammation is to be noticed and shown by the presence of large numbers of leucocytes, while red cells are found scattered in the interstitial tissue; at some places small hemorrhages can be seen.

The changes in the glomeruli are principally confined to a thickening in Bowman's capsule, the tuft of bloodvessels having as a whole remained intact.

The tubules in the medullary part of the organ seemed to have escaped the destructive changes seen elsewhere, although the epithelium has a slightly cloudy and granular appearance.

The bloodvessels show a marked thickening of their tunica media and a softening or breaking down of the intima. I remember of seeing in one place a complete detachment of the inner coat from the middle one, but I am inclined to take this for an artefact.

---

Does it not sound inconsistent for a man with horse sense to purchase an automobile.—*Rural New Yorker*.

An outbreak of foot-and-mouth disease among the livestock of Argentina has caused the English Board of Agriculture to prohibit the importation of South American animals into English ports.

